# Inhibition of β-Catenin to Overcome Endocrine Resistance in Tamoxifen-Resistant Breast Cancer Cell Line

**DOI:** 10.1371/journal.pone.0155983

**Published:** 2016-05-19

**Authors:** Hye Sung Won, Kyung Mee Lee, Ju Eon Oh, Eun Mi Nam, Kyoung Eun Lee

**Affiliations:** 1 Division of Medical Oncology, Department of Internal Medicine, College of Medicine, The Catholic University of Korea, Seoul, Korea; 2 Division of Hematology-Oncology, Departments of Internal Medicine, Ewha Medical Research Center, School of Medicine, Ewha Womans University, Seoul, Korea; II Università di Napoli, ITALY

## Abstract

**Background:**

The β-catenin signaling is important in cell growth and differentiation and is frequently dysregulated in various cancers. The most well-known mechanism of endocrine resistance is cross-talk between the estrogen receptor (ER) and other growth factor signaling, such as phosphatidylinositol-3-kinase (PI3K)/Akt and the mammalian target of rapamycin (mTOR) signaling pathway. In the present study, we investigated whether β-catenin could be a potential target to overcome endocrine resistance in breast cancer.

**Methods:**

We established tamoxifen-resistant (TamR) cell line via long-term exposure of MCF-7 breast cancer cells to gradually increasing concentrations of tamoxifen. The levels of protein expression and mRNA transcripts were determined using western blot analysis and real-time quantitative PCR. The transcriptional activity of β-catenin was measured using luciferase activity assay.

**Results:**

TamR cells showed a mesenchymal phenotype, and exhibited a relatively decreased expression of ER and increased expression of human epidermal growth factor receptor 2 and the epidermal growth factor receptor. We confirmed that the expression and transcriptional activity of β-catenin were increased in TamR cells compared with control cells. The expression and transcriptional activity of β-catenin were inhibited by β-catenin small-molecule inhibitor, ICG-001 or β-catenin siRNA. The viability of TamR cells, which showed no change after treatment with tamoxifen, was reduced by ICG-001 or β-catenin siRNA. The combination of ICG-001 and mTOR inhibitor, rapamycin, yielded an additive effect on the inhibition of viability in TamR cells.

**Conclusion:**

These results suggest that β-catenin plays a role in tamoxifen-resistant breast cancer, and the inhibition of β-catenin may be a potential target in tamoxifen-resistant breast cancer.

## Introduction

Breast cancer is the second most common malignancy among women in South Korea. It is a heterogeneous disease that can be classified into multiple subtypes with distinctive histological and biological features [1]. The most common subtype is the hormone receptor-positive breast cancer, about 70–75% of all breast cancers express the estrogen receptor (ER) or progesterone receptor (PR) [2]. Therefore, endocrine therapy to block ER activity is an important treatment for these patients [2]. Tamoxifen, which is a selective ER modulator, has been the mainstay of endocrine therapy for the management of ER-positive breast cancer. However, de novo (primary) or acquired (secondary) resistance to endocrine therapy remains an important clinical issue. About 20–30% of patients who received adjuvant tamoxifen experience relapse, and the majority of patients with advanced disease who showed an initial good response to tamoxifen eventually experience disease progression [3]. Thus, acquired resistance to endocrine therapy is common in clinical practice, and overcoming this resistance remains a crucial challenge in the treatment of ER-positive breast cancer.

Over the past few decades, there have been many studies about the mechanisms of resistance to endocrine therapy. Although the exact molecular mechanisms underlying this phenomenon are still not completely understood, several theories have been proposed, such as the loss of ER expression, mutations within the gene that encodes the ER, adaptation of estrogen withdrawal, cross-talk with other growth factor receptor pathways, and alteration of the cell-cycle signaling pathway [2, 4, 5]. Actually, about 20% of patients treated with endocrine therapy show a loss of ER in tumors over time [5]. These tumors would no longer be driven by ER, and other pathways may adopt for the role of oncogenic driver. To date, the most well-known alternatively activated pathway is the phosphatidylinositol-3-kinase (PI3K)/Akt and the mammalian target of rapamycin (mTOR) signaling pathway [2].

Aberrant activation of Wnt/β-catenin signaling is observed in many human cancers, such as colon cancer [6]. Recent studies of breast cancer suggested that activation of β-catenin signaling is enriched in the triple-negative phenotype without ER expression and is associated with poor outcome [7]. Therefore, we concerned about whether β-catenin signaling as an alternative pathway for endocrine resistance in breast cancer. The β-catenin is important in developmental processes, cell growth, differentiation, invasion, and survival. Inactivation of β-catenin signaling leads to the formation of the "destruction complex", which consists of adenomatous polyposis coli, Axin, glycogen synthase kinase-3β (GSK-3β) and casein kinase 1α. This "destruction complex" phosphorylates β-catenin; phosphorylated β-catenin is then targeted for ubiquitination and proteolytic degradation [8]. Conversely, the binding of Wnt ligands to receptors prevents the GSK3β-dependent phosphorylation of β-catenin and leads to its stabilization. Stabilized β-catenin proteins translocate into the nucleus and interact with the T-cell factor (TCF)/lymphocyte enhancer factor (LEF). The β-catenin/TCF complex regulates the transcription of many target genes that are associated with cell proliferation in cancer [8].

In this study, we aimed to assess the expression and transcriptional activity of β-catenin in tamoxifen-resistant breast cancer cell line and evaluate the effect of inhibition of β-catenin on the viability of tamoxifen-resistant breast cancer cells.

## Materials and Methods

### Cell lines and cell culture

The human breast cancer cell line MCF-7 was purchased from the Korean Cell Line Bank (Seoul, South Korea). MCF-7 cells are a well-characterized ER-positive control cell line. MCF-7 cells were seeded at a density of 2 × 10^5^/cm^2^ and cultured in phenol-red-free RPMI 1640 medium containing 10% fetal bovine serum (FBS) and antibiotics. According to a methodology reported elsewhere [9], we established an MCF-7-derived tamoxifen-resistant cell line (TamR) via long-term culture of MCF-7 cells in the presence of 4-hydroxytamoxifen (Sigma-Aldrich, St. Louis, MO, USA). Briefly, MCF-7 cell monolayers were washed with phosphate-buffered saline (PBS) and transferred to phenol-red-free RPMI 1640 medium containing 10% charcoal-stripped, steroid-depleted FBS (Sigma-Aldrich), antibiotics, and 4-hydroxytamoxifen (10^−7^ M in ethanol). The cells were exposed to this treatment for 1 week, during which the medium was replaced twice a week. To generate drug-resistant cell lines, the cells were cultured in the presence of gradually increasing concentrations of 4-hydroxytamoxifen from 0.05 to 3 μM over a period of 8 months.

### Cell viability assay

Cell viability was measured using the EZ-Cytox Cell Viability Assay kit (Daeil Lab Service, Seoul, South Korea). Briefly, MCF-7 and TamR cells were seeded into 96-well plates at a density of 2 × 10^3^ cells/well and then cultured in a CO_2_ incubator (5% CO_2_, 37°C) for 24 hours. To evaluate dose-dependent effect, cells were treated with each concentration of 4-hydroxytamoxifen (0, 3, 6, and 9 μM) for 24 hours, and then 10 μL of EZ-Cytox solution was added to each well of the plate. After 30 minutes of incubation, the plates were read at 560 nm on a spectrophotometer. A cell viability assay was performed in the same way after ICG-001 (Selleckchem, Houston, TX, USA) or rapamycin (Sigma-Aldrich) treatment for 24 hours. ICG-001 is a small-molecule inhibitor of β-catenin and rapamycin is a well-known inhibitor of the serine/threonine protein kinase mTOR.

### RNA isolation and real time PCR

MCF-7 and TamR cells were seeded at a density of 1 × 10^5^ cells/mL in 12-well plates. On the following day, cells were serum starved for 16 hours and then cultured in RPMI 1640 medium supplemented with 10% FBS for 24 hours. RNA was extracted from MCF-7 and TamR cells using the RNeasy mini kit (Qiagen, Valencia, CA, USA), according to the manufacturer’s instructions, and was quantitatively analyzed on a nanodrop spectrophotometer. cDNA was synthesized using 500 ng of total RNA and the cDNA Synthesis Kit (iNtRON Biotechnology, Gyeonggi-do, South Korea) at the following temperatures: 60 minutes at 45°C, 5 minutes at 95°C, and 5 minutes at 4°C. Real-time PCR (RT–PCR) was performed to quantify the mRNA of the ER-alpha (ERα), human epidermal growth factor receptor 2 (HER2), and epidermal growth factor receptor (EGFR). A reaction mixture (12 μL) consisting of 10 ng of diluted cDNA preparation (2 μL), the SYBR Green Dye (Qiagen, Valencia, CA, USA), and 10 pmol of each primer (0.5 μL) was used for PCR using the following thermal cycler conditions: denaturation, 95°C for 5 minutes; amplification, 45 cycles of 95°C for 10 seconds, 60°C for 10 seconds, and 72°C for 10 seconds. The analysis was repeated three times for each sample and the relative expression levels of ERα, EGFR, and HER2 were normalized to that of glyceraldehyde-3-phosphate dehydrogenase.

### Western blot analyses

MCF-7 and TamR cells were seeded at a density of 1 × 10^5^ cells/mL in 100 mm dishes. On the following day, cells were serum starved for 16 hours and were then incubated with rapamycin, ICG-001, and a combination of rapamycin and ICG-001 in RPMI 1640 medium supplemented with 1% FBS for 24 hours. The separation and quantification of proteins were performed with the PRO-PREPTM protein extraction solution (iNtRON Biotechnology). Briefly, cells were lysed in 500 μL of ice-cold PRO-PREP lysis buffer for 20 minutes. Lysates were centrifuged at 13,000 rpm for 10 minutes at 4°C, and the supernatants were collected. Total protein concentrations were determined using the Bradford assay (Bio-Rad, Hercules, CA, USA) using a spectrophotometer at 595 nm. A total of 50 μg of protein was mixed with the 5× sample buffer and boiled for 5 minutes. Proteins were separated using 10% sodium dodecyl sulfate–polyacrylamide gel electrophoresis (SDS–PAGE) and transferred to polyvinylidene difluoride membranes. Membranes were blocked in 5% skim milk-Tris-buffered saline (TBS)-Tween for 1 hour and then incubated with shaking for 2 hours in primary antibodies (1:1,000 dilution). Blots were washed with TBS-Tween buffer and then incubated for 1 hour with an anti-rabbit-Horseradish Peroxidase-conjugated secondary antibody (1:1,000 dilution). Immunoblots were treated with enhanced chemiluminescence (ECL) reagents and visualized on a LAS luminescent image analyzer (Fujifilm Life Science). All antibodies were purchased from Cell Signaling Technology (Beverly, MA, USA): the antibodies used were against ERα, HER2, EGFR, Akt, phospho-Akt (Ser473, pAkt), GSK-3β, phospho-GSK-3β (Ser9, pGSK-3β), mitogen-induced p70 ribosomal protein S6 kinase (p70S6K), phospho-p70S6K (Thr389, pp70S6K), β-catenin, nonphospho (active) β-catenin, cyclin D1 (cat #2978), E-cadherin, N-cadherin, Snail, Slug, Twist, and c-myc. All of these are rabbit monoclonal antibodies.

### Pull-down assay

We prepared Luria–Bertani (LB) media containing the antibiotic ampicillin for the growth of bacteria. The glutathione S-transferase (GST)-E-cadherin plasmid was kindly provided by Professor Stuart Aaronson, Oncological Sciences, Mount Sinai Hospital in New York, USA. The GST-E-cadherin plasmid was transformed into the BL21 strain of E. coli. For the induction of protein, isoprolyl-β-D-thio-galactopyranoside (IPTG) was added to a final concentration of 0.1 mM. After extraction, proteins were bound on glutathione-Sepharose resin for 1 hour. The glutathione-Sepharose resin attached to GST-E-cadherin protein was incubated with proteins extracted from control and TamR cells for 1 hour. After centrifugation and removal of the supernatant, beads were eluted using a 10 mM glutathione elution butter. After incubation at room temperature for 10 minutes, centrifugation was performed and the supernatant was transferred into a new tube. The proteins were analyzed by SDS–PAGE and western blotting.

### Luciferase assay

Luciferase assay kit from Promega (Madison, WI, USA) was used. MCF-7 and TamR cells were seeded at a density of 4 × 10^4^ cells/0.5 mL/well in 24-well plates the day before transfection. Cells were serum starved for 16 hours and then cotransfected with the pGL4.49 [luc2p/TCF-LEF RE/Hygro] (Promega) and pRL-TK constructs (Promega) using Lipofectamine 2000 (Invitrogen Korea, Seoul, South Korea). The pGL4.49 [luc2p/TCF-LEF RE/Hygro] vector contains eight copies of a TCF-LEF response element (TEF-LEF RE) that drives transcription of the firefly luciferase reporter gene luc 2p. The pRL-TK vector provides constitutive expression of Renilla luciferase as a control reporter vector. The cells were incubated for 24 hours after transfection, followed by treatment with ICG-001 or rapamycin for 24 hours. The cells were then processed using the DUAL-Luciferase Reporter Assay System (Promega), and luciferase activities were measured on a luminometer. The ratio of firefly to Renilla luciferase activity was representative of the transcriptional activity of β-catenin.

### Transfection and knockdown

The siRNA against β-catenin was obtained from Cell Signaling Technology (SignalSilence^®^ β-catenin siRNA I). The mixture of β-catenin siRNA (100 nM) and Lipofectamine 2000 in Opti-MEM was incubated in room air for 20 minutes. MCF-7 and TamR cells were seeded at a density of 5 × 10^5^ cells in 100 mm dishes and then were incubated with mixture of β-catenin siRNA and Lipofectamine 2000 in a CO2 incubator (5% CO2, 37°C) for 48 hours. The Control siRNA (SignalSilence^®^ Control (unconjugated) siRNA) was used as negative control (Mock).

### Flow cytometric analysis of cell cycle

Cells were treated with ICG001 (40μM) and rapamycin (20nM) for 24 h for cell cycle analysis. The cells were detached with trypsin and pelleted by centrifugation at 1,200 rpm for 5 min, followed by fixation with 70% ethanol for overnight at -20°C. The cells were washed with PBS and cellular DNA was stained with 0.5 ml of protium iodide (PI) solution (BD, 50 ug/ml propium iodide, 5 mM EDTA and 1 mg/ml RNase in PBS) for 30 min at room temperature. For annexin V staining, cells were stained with FITC annexin V in binding buffer (10 mM HEPES (pH 7.4), 140 mM NaCl, 0.25 mM CaCl2) and 5 ug/ml of PI. Analytical cytometry was performed on FACSCalibur (BD). Cell cycle analysis was performed with CellQuest (BD) and ModFit (Verity, Topsham, ME) software.

### Statistical analysis

Results were presented as means with error bars representing the standard deviation. Statistical analyses were performed using SPSS (IBM Company, version 18). An unpaired t-test was used to evaluate significant differences among the continuous variables. A P-value ≤ 0.05 was considered statistically significant.

## Results

### Characteristics of tamoxifen-resistant breast cancer cell lines

**Fig 1:**
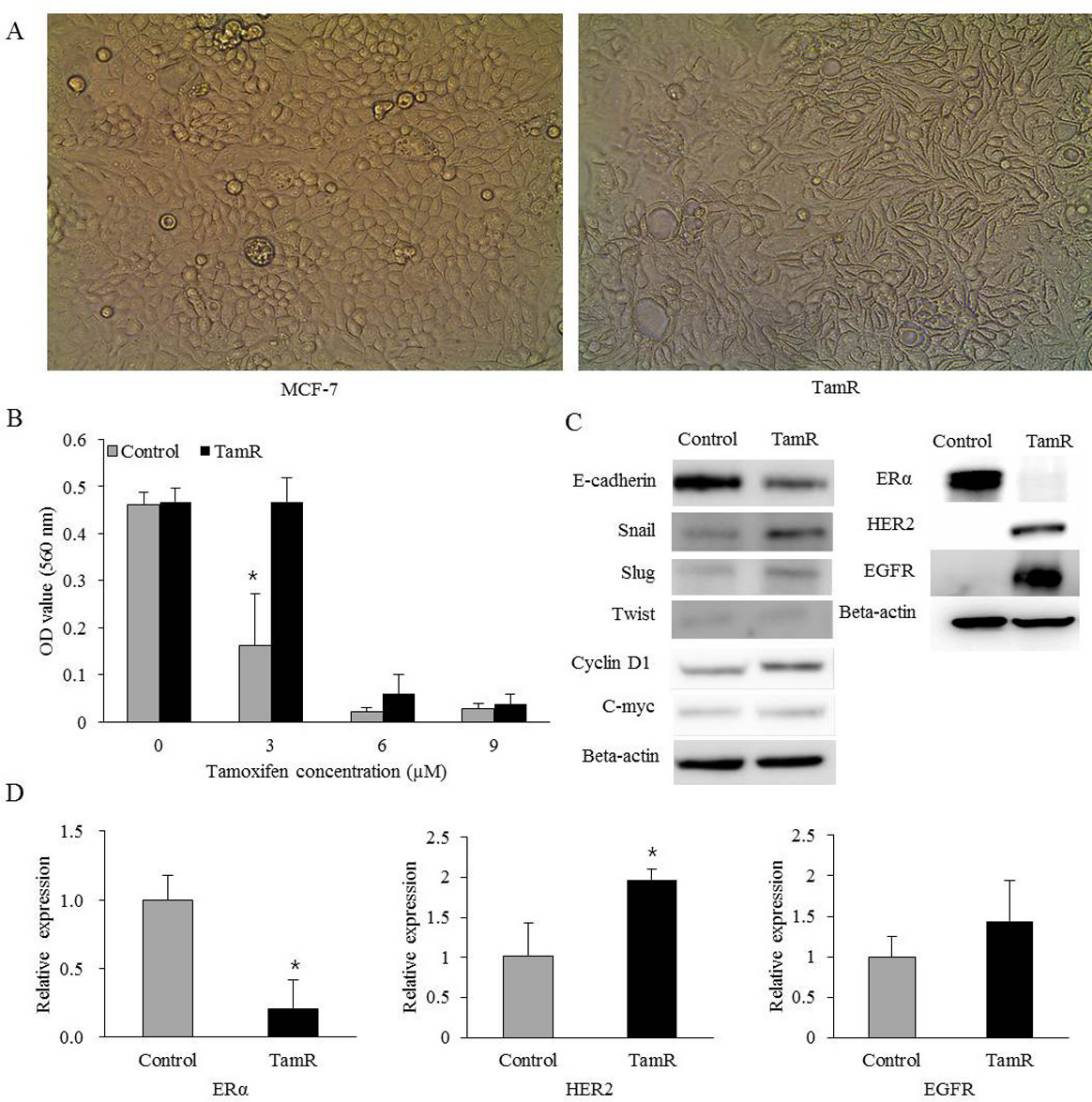
Characteristics of tamoxifen-resistant breast cancer cell lines. (A) TamR cells exhibited a more elongated, spindle-shaped morphology, whereas control cells exhibited a cuboidal shape (magnification 100×). (B) The viability of control cells decreased by 65.2% after 3 μM tamoxifen treatment, whereas the viability of TamR cells showed no change. (C) Western blot analysis of EMT-related markers, estrogen receptor alpha (ERα), human epidermal growth factor receptor 2 (HER2) and epidermal growth factor receptor (EGFR) in control and TamR cells. TamR cells showed decreased expression of E-cadherin and increased expression of Snail, Slug, and cyclin D1. TamR cells showed decreased expression of ERα and increased expression of HER2 and EGFR. (D) mRNA expression of ERα, HER2 and EGFR was assessed by RT-PCR. TamR cells exhibited a relatively decreased expression of ERα and increased expression of HER2 and EGFR. Error bars, mean ± standard deviation (SD). *, P < 0.05.

TamR cells had a markedly different morphology compared with the parental control cells (Fig 1A). The majority of control cells exhibited a cuboidal shape, whereas TamR cells exhibited a more spindle-shaped morphology, which was similar to that of mesenchymal cells. The cell viability assay was performed according to dose of tamoxifen (Fig 1B). TamR and control cells were treated with various doses of tamoxifen. The viability of control cells decreased by 65.2% after 3 μM tamoxifen treatment, whereas the viability of TamR cells showed no change. Thus, TamR cells exhibited resistance to tamoxifen. Because the morphological changes which were shown in TamR cells, were associated with epithelial-mesenchymal transition (EMT) phenotype, we proceeded to assess the expression of EMT-related markers using western blotting. TamR cells exhibited E-cadherin loss and increased expression of Snail, Slug, and cyclin D1 (Fig 1C). There was no difference in the expression of Twist and c-myc between control and TamR cells. The expression of N-cadherin was not observed in both control and TamR cells (data not shown). The transcript and protein expression levels of ERα, HER2 and EGFR were determined using RT–PCR and western blot analysis. The relative expression of ERα was significantly decreased in TamR cells compared with control cells. On the other hand, the relative expression of HER2 and EGFR was increased in TamR cells compared with control cells, respectively (Fig 1C and 1D).

### Increased expression and activity of β-catenin and its inhibition in tamoxifen-resistant breast cancer cell lines

**Fig 2:**
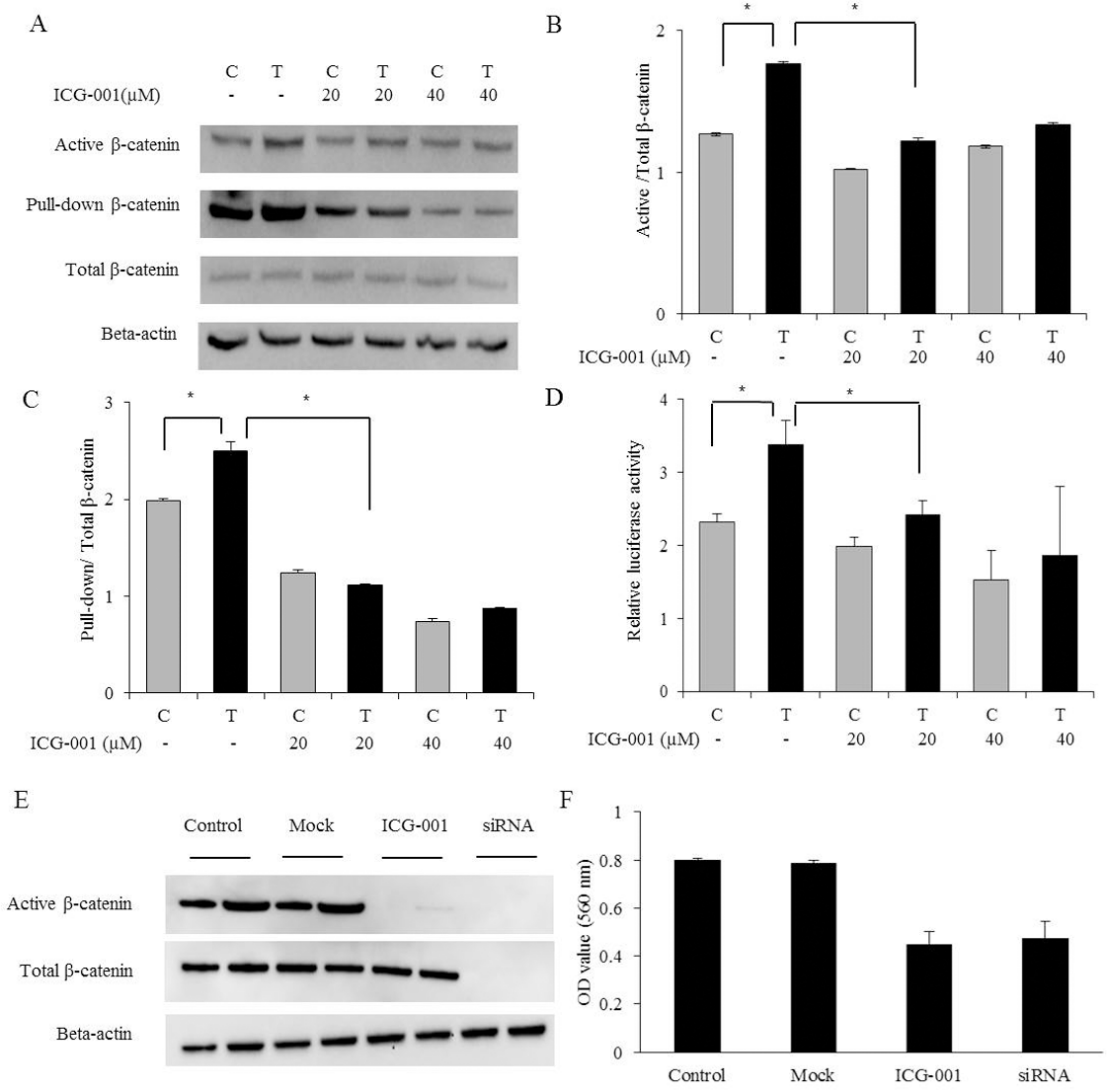
Protein expression and transcriptional activity of β-catenin and its inhibitory effects in TamR cells. (A) Protein expression levels of β-catenin were measured by western blot analysis using a commercial primary antibody and pull-down assay. The expression of β-catenin was increased in TamR cells and was inhibited by ICG-001. (B) Quantification of band intensities was determined by densitometry analysis. The graphs showed the active β-catenin divided by total β-catenin. (C) Quantification of band intensities was determined by densitometry analysis. The graphs showed the pull-down β-catenin divided by total β-catenin. (D) The transcriptional activity of β-catenin was assessed using luciferase reporter assay. The transcriptional activity of β-catenin was increased in TamR cells and was inhibited by ICG-001. (E) The protein expression of β-catenin was also inhibited by β-catenin siRNA. (F) Cell viability assay of TamR cells was assessed after treatment with ICG-001 (40 μM) and β-catenin siRNA for 24 hours. The viability of TamR cells was decreased by ICG-001 and β-catenin siRNA. The experiment was repeated three times. Error bars, mean ± SD. *, P < 0.05. C, control cells. T, TamR cells.

We confirmed the protein expression levels of unphosphorylated transcriptionally active β-catenin using two methods: Western blot analysis using a commercial primary antibody and pull-down assay. Both methods showed that the expression of the active β-catenin protein was increased in TamR cells compared with control cells (Fig 2A–2C). The transcriptional activity of β-catenin was confirmed using a luciferase reporter assay. TamR cells showed a significantly increased β-catenin transcriptional activity compared with control cells (Fig 2D).

ICG-001 is a small-molecule inhibitor that antagonizes β-catenin/TCF-mediated transcription [10]. Transcriptional regulation of the β-catenin/TCF complex requires some coactivators, such as the cAMP response element-binding protein (CREB)-binding protein (CBP). ICG-001 specifically binds to CBP, resulting in the prevention of the interaction between β-catenin and CBP [10]. We measured the protein levels and transcriptional activity of active β-catenin after treatment with ICG-001 for 24 hours in TamR and control cells. ICG-001 significantly reduced the protein levels and transcriptional activity of active β-catenin. This effect was more pronounced in TamR cells compared with control cells (Fig 2A–2D).

To the next, control and TamR cells were transfected with siRNA specific for β-catenin and then the protein levels of β-catenin were assessed by western blot analysis. The β-catenin siRNA suppressed the expression of total and active β-catenin (Fig 2E). Taken together, the expression of active β-catenin was decreased with both ICG-001 and siRNA.

Cell viability assays were conducted to determine the growth inhibitory effect of inhibition of β-catenin. TamR cells did not show a change in viability by tamoxifen (3 μM). However, ICG-001 (40 μM) reduced the viability of TamR cells by 45% and β-catenin siRNA also reduced the viability of TamR cells by 41% (Fig 2F).

### Combinatory effect of β-catenin and mTOR inhibition in tamoxifen-resistant breast cancer cell lines

**Fig 3:**
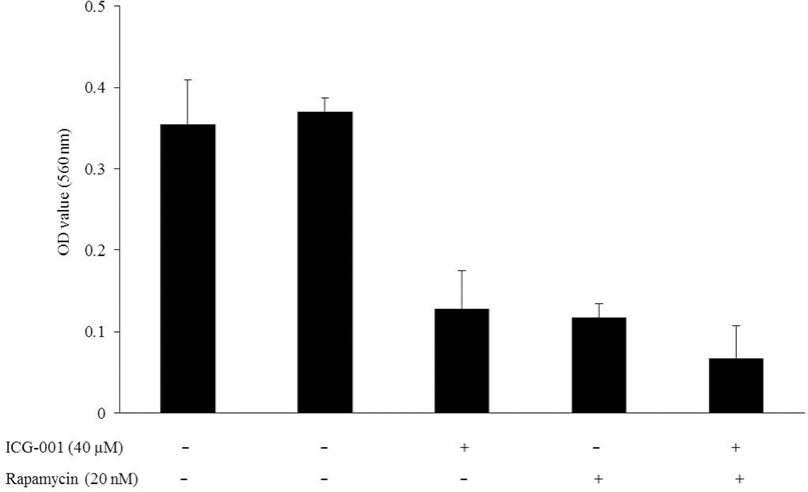
Combinatory effect of ICG-001 and rapamycin in TamR cells. Cell viability assay of TamR cells after treatment with ICG-001 (40 μM) and rapamycin (20 nM) for 24 hours was performed. The viability of TamR cells, which had showed resistance to tamoxifen (3 μM), was decreased by ICG-001 and rapamycin. All media except first lane were treated with tamoxifen (3 μM).

PI3K/Akt/mTOR signaling pathway is a well-known mechanism of endocrine resistance. Rapamycin is the mTOR inhibitor, which previously showed overcoming endocrine resistance in breast cancer cells [11]. To confirm the dual inhibitory effects of β-catenin and mTOR in endocrine resistance, we performed cell viability assay after treatment of rapamycin and a combination of ICG-001 and rapamycin, respectively. The viability of TamR cells showed no change after 3 μM tamoxifen treatment. Rapamycin (20 nM) reduced the viability of TamR cells by 68.3%. The combination of ICG-001 (40 μM) and rapamycin (20 nM) reduced the viability of TamR cells by 81.7% (Fig 3). To analyze the combined effects of these drugs, we calculated combination index (CI) values for different concentrations at a constant ratio using the CalcuSyn software (Biosoft, Cambridge, U.K.). A CI between 0.9 and 1.1 indicates an additive effect, and a CI ≤ 0.9 indicates synergy [12]. After combination treatment of TamR cells with ICG-001 and rapamycin, the CI values at a fractional effect analysis value of 0.5, 0.75, and 0.9 were 1.314, 1.108, and 1.022, respectively. Thus, we found an additive effect of the two drugs, ICG-001 and rapamycin, at higher concentrations in TamR cells.

**Fig 4:**
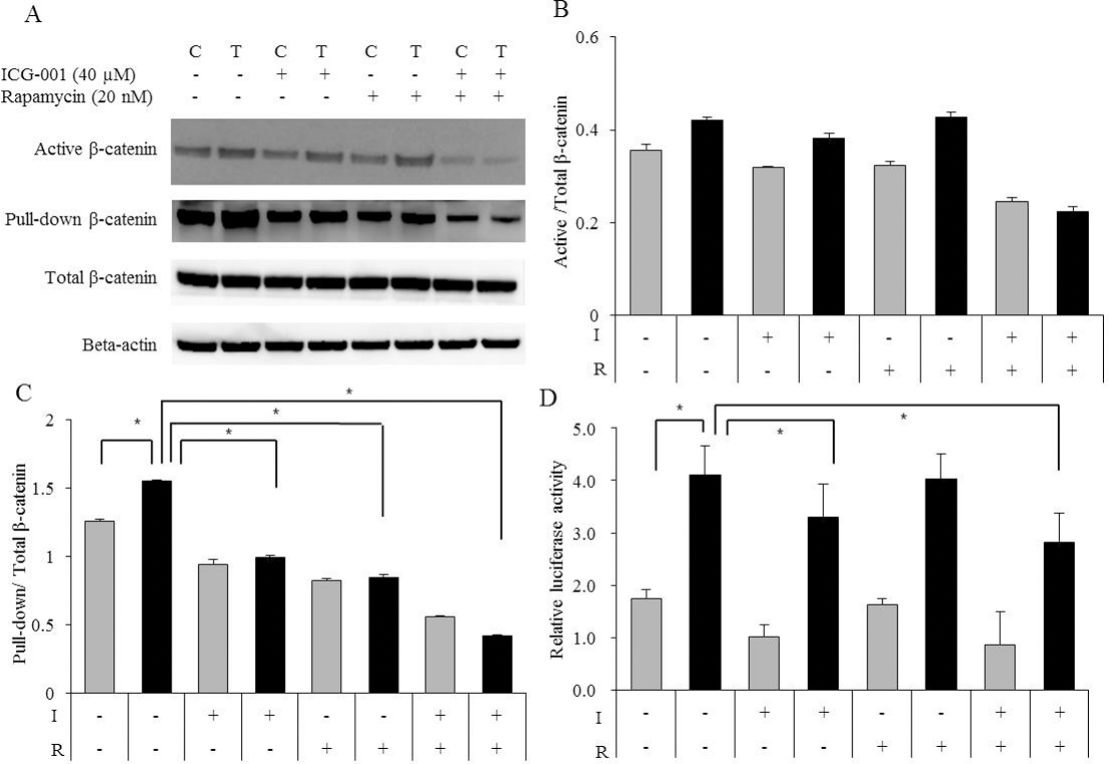
Alteration of β-catenin after treatment with ICG-001 and rapamycin. (A) The expression of β-catenin was decreased by ICG-001 and rapamycin. It was decreased to a greater extent by the combination of ICG-001 and rapamycin. (B) Quantification of band intensities was determined by densitometry analysis. The graphs showed the active β-catenin divided by total β-catenin. (C) Quantification of band intensities was determined by densitometry analysis. The graphs showed the pull-down β-catenin divided by total β-catenin. (D) The transcriptional activity of β-catenin reduced to the greatest extent by the combination of ICG-001 and rapamycin. The experiment was repeated three times. Error bars, mean ± SD. *, P < 0.05. I, ICG-001 (40 μM). R, Rapamycin (20 nM). C, control cells. T, TamR cells.

We evaluated the protein levels and transcriptional activity of active β-catenin after treatment with ICG-001 (40 μM), rapamycin (20 nM), and a combination of ICG-001 and rapamycin, respectively. The expression β-catenin was decreased by rapamycin as well as by ICG-001. Pull-down assays tend to measure the expression of β-catenin more accurately. The combination of ICG-001 and rapamycin led to a greater decrease in the expression and transcriptional activity of β-catenin compared with each single inhibitor (Fig 4A–4D).

**Fig 5:**
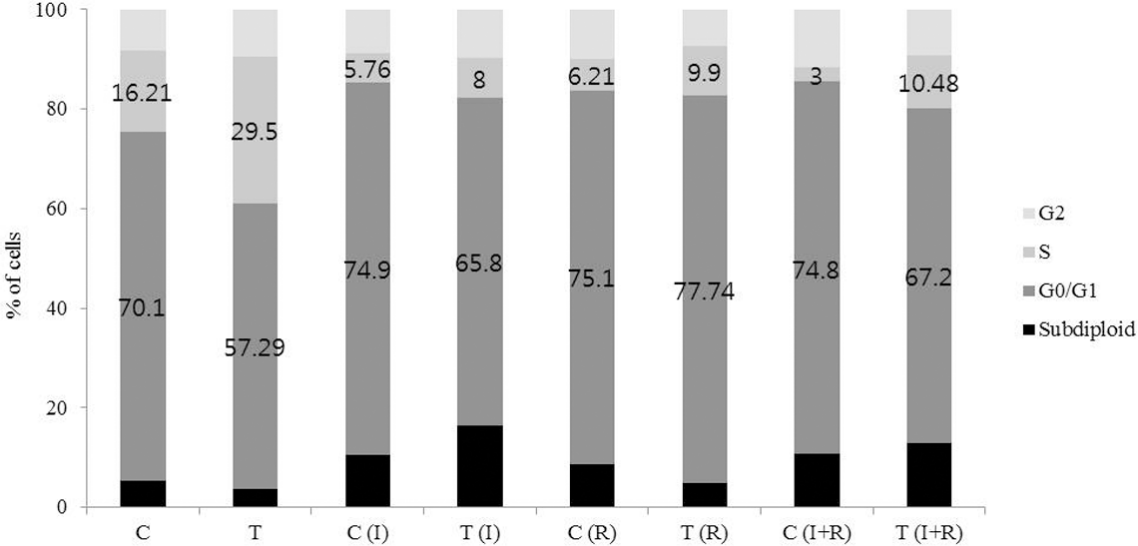
Cell cycle analysis of control and TamR cells with ICG-001 and rapamycin. Flow cytometric analysis of cell cycle was performed in control and TamR cells after treatment with ICG-001 (40 μM) and rapamycin (20 nM) for 24 hours. TamR cells showed the accelerated G1 to S phase transition. ICG-001 increased the fraction of apoptotic cells and rapamycin increased the fraction of G0/G1-arrested cells. I, ICG-001 (40 μM). R, Rapamycin (20 nM). C, control cells. T, TamR cells.

Cell cycle of control and TamR cells was analyzed by flow cytometry. The results showed that TamR cells had an acceleration of the G1 to S phase transition compared to control cells, indicating that cell cycle progression was promoted in TamR cells. ICG-001 increased apoptosis and rapamycin induced significant G0/G1 phase arrest in TamR cells (Fig 5). The combination of ICG-001 and rapamycin seems to exert its growth-inhibitory effect on the TamR cells by both cell cycle arrest and apoptosis.

**Fig 6:**
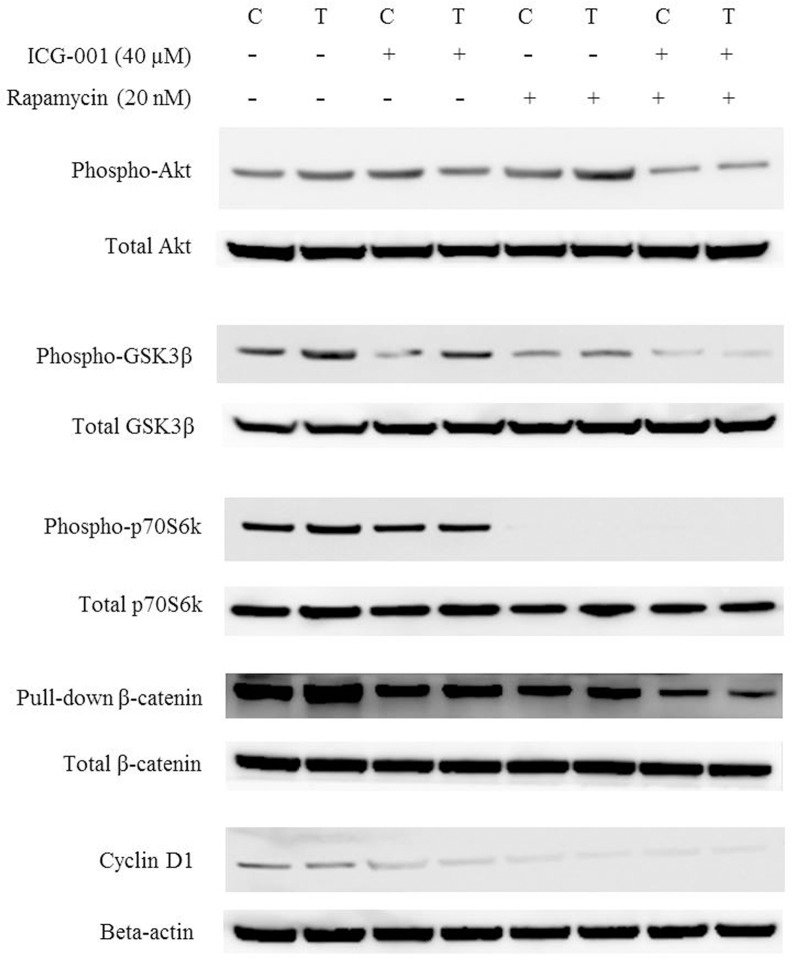
Alteration of PI3K/Akt/mTOR and β-catenin pathway- related proteins after treatment with ICG-001 and rapamycin.

We confirmed the protein levels of pAkt, pGSK-3β, and pp70S6K in TamR cells, as well as the change in their expression levels after treatment with ICG-001 (40 μM), rapamycin (20 nM), and a combination of ICG-001 and rapamycin (Fig 6). The expression of pAkt, pGSK-3β, and pp70S6K was slightly increased in TamR cells compared with control cells. This suggests that Akt/GSK-3β/mTOR signaling is activated in TamR cells. Rapamycin blocked p70S6K phosphorylation and activation. It caused a slight increase in pAkt levels and slight decrease in pGSK-3β levels because of the loss of the p70S6K-mediated negative feedback on Akt and GSK-3β. ICG-001 also slightly downregulated pGSK-3β in TamR cells and it may affect the Akt/mTOR signaling. Treatment with a combination of rapamycin and ICG-001 led to a blockage of p70S6K phosphorylation that was similar to that observed for the rapamycin treatment. However, pAkt and pGSK-3β levels were decreased to a greater extent, which was different from that observed for the rapamycin treatment. We confirmed the expression of cyclin D1, which is a target gene of ERα, β-catenin, and mTOR. The expression level of cyclin D1 in TamR cells was comparable with that of control cells, and was significantly decreased by ICG-001 and rapamycin (Fig 6). There was no significant change in expression level of c-myc between control and TamR cells (data not shown).

## Discussion

We established tamoxifen-resistant breast cancer cell line by continuously exposing ER-positive breast cancer cells to tamoxifen by a gradual process. In the present study, TamR cells showed relatively decreased expression of ERα and increased expression of HER2 and EGFR. This result was consistent with previous research [13] and indicated that other signaling pathways, rather than ERα signaling, are involved in TamR cells, the most widely known among them being the PI3K/Akt/mTOR signaling pathway [2, 14]. In addition to PI3K/Akt/mTOR signaling, we were interested in the role of β-catenin in endocrine resistance. The results demonstrated that the expression and transcriptional activity of β-catenin were increased in TamR cells and were effectively inhibited by ICG-001 or β-catenin siRNA. To obtain insight into overcoming endocrine resistance via the inhibition of β-catenin, we measured changes in the viability of TamR cells after inhibition of β-catenin. TamR cells showed no change in cell viability after treatment of tamoxifen, but ICG-001 or β-catenin siRNA reduced the viability of TamR cells. This suggests that β-catenin plays a role in endocrine resistance, and that inhibition of β-catenin is another therapeutic target for patients with breast cancer who have tamoxifen resistance.

The previous studies have reported that activation of the PI3K/Akt/mTOR pathway is associated with endocrine resistance, and that sensitivity to endocrine therapy might be reversed by inhibition of this pathway [14–16]. deGraffenried et al reported that the inhibition of mTOR restored tamoxifen sensitivity in breast cancer cells with aberrant Akt activity [11]. According to their study, MCF-7 breast cancer cells that express a constitutively active Akt showed resistance to tamoxifen, however, treatment with rapamycin inhibited the growth of resistant cells by 65%, as shown by a cell viability assay. They suggested that cotreatment with rapamycin restored tamoxifen response in breast cancer cells with aberrant Akt activity [11]. The present study yielded similar results. The expression of pAkt and pp70S6K, which is main downstream effectors of mTOR, was slightly increased in TamR cells. Treatment of TamR cells with tamoxifen did not change cell viability. However, the addition of rapamycin reduced the viability of TamR cells by 68.3%. Also, the combination of ICG-001 and rapamycin reduced the viability of TamR cells by 81.7%. Taken together, inhibition of β-catenin had the effect on the inhibition of the growth as much as rapamycin in TamR cells. The combination of ICG-001 and rapamycin had an additive effect on the inhibition of the growth of TamR cells. Cell cycle analysis was performed to confirm whether growth inhibitory effects of ICG-001 and rapamycin are due to cell cycle arrest or apoptosis. We found that TamR cells showed the accelerated G1 to S phase transition. ICG-001 induced apoptosis of cells and rapamycin led to prolonged G1 phase in TamR cells. It suggests that the growth inhibitory effect of ICG-001 and rapamycin on TamR cells is due to both cell cycle arrest and apoptosis.

To the next, studies on the altered expression of β-catenin and mTOR-related molecules were performed to identify a mechanism of action and interaction between β-catenin and mTOR pathways. Cyclin D1 is a well-known regulator of cell-cycle progression, and a target gene of the ERα, β-catenin, and mTOR [8, 17, 18]. Estrogen induces the expression of cyclin D1 in ER-positive breast cancer cells, and treatment with tamoxifen downregulates the expression of cyclin D1 [19]. Aberrant expression of cyclin D1, despite the presence of tamoxifen, has been shown to lead to endocrine-resistant cell growth [20]. In the present study, the expression of cyclin D1 was maintained consistently in TamR cells, although the expression of the ERα was suppressed significantly. The expression of cyclin D1 was decreased significantly after treatment with ICG-001 and/or rapamycin. This suggests that other pathways that work to overcome tamoxifen-induced cell-cycle arrest are activated in TamR cells, possibly the mTOR and β-catenin pathways. Thus, blocking these pathways may induce cell-cycle arrest again, resulting in the overcoming of endocrine resistance.

Several recent studies reported a relationship between PI3K/Akt/mTOR and β-catenin signaling [21–23]. Growth factors activate PI3K, which subsequently activates Akt via phosphorylation. The phosphorylated Akt activates mTOR, which subsequently phosphorylates p70S6K, leading to the transcription of target genes related to cell proliferation [24]. GSK-3β, which is inactivated by phosphorylation at Ser9, negatively regulates mTOR. Various kinases, such as active Akt and p70S6K, can phosphorylate GSK-3β at Ser9, resulting in the inactivation of GSK-3β [25]. On the other hand, Wnt stimulation generally induces the accumulation of β-catenin by inhibiting the formation of the destruction complex. However, overexpression of β-catenin is also induced by Wnt-independent signaling; i.e., the phosphorylation of GSK-3β by activated Akt can result in a substantial decrease in the formation of the destruction complex of β-catenin, thereby leading to increased accumulation of β-catenin [24, 26, 27]. Therefore, it may be possible to modulate the interaction between PI3K/Akt/mTOR and the β-catenin pathway by mediating GSK-3β. In the present study, rapamycin, which completely blocked the activation of p70S6K, increased the expression of pAkt and decreased the expression of pGSK-3β. This was caused by the elimination of the p70S6K negative feedback on Akt and GSK-3β. This result suggests that rapamycin activates GSK-3β [21], thereby decreasing the β-catenin via the destruction complex. Our study showed that ICG-001 led to downregulation of pGSK-3β. Hao YQ et al. also showed that the expression of pGSK-3β was decreased by ICG-001 compared with control in human alveolar epithelial cell line A549 [28]. The mechanism of the effect of ICG-001 on GSK-3β phosphorylation is unclear. This result suggests that GSK-3β is activated in the process of inhibition of β-catenin by ICG-001, which may have affected the Akt/mTOR signaling.

The activity of GSK-3β and β-catenin were decreased to the greatest extent by the combination of ICG-001 and rapamycin. The exact mechanisms of interaction between PI3K/Akt/mTOR and the β-catenin pathway are not yet fully understood, and further study is needed for this.

In summary, the results of this study suggest that the β-catenin pathway plays a considerable role in breast cancer with acquired tamoxifen-resistance. It is expected that the inhibition of β-catenin will be act as a new targeted therapy in endocrine resistance, such as mTOR inhibitor.
